# Low-Dose Rivaroxaban vs. Aspirin in Addition to Clopidogrel After Percutaneous Coronary Intervention in Coronary Atherosclerotic Heart Disease Patients with Gastrointestinal Disease

**DOI:** 10.1007/s10557-025-07682-5

**Published:** 2025-03-21

**Authors:** Yue Li, Tienan Zhou, Yan Liu, Junxian Qi, Lei Zhang, Ruoxi Gu, Dongyuan Sun, Xiaozeng Wang

**Affiliations:** 1 National Key Laboratory of Frigid Zone Cardiovascular Disease, Cardiovascular Research Institute and Department of Cardiology, General Hospital of Northern Theater Command, Shenyang, Liaoning 110016 China; 2 College of Life Science and Biopharmaceutical, Shenyang Pharmaceutical University, Shenyang, Liaoning 110016 China

**Keywords:** Rivaroxaban, Low dose, Gastrointestinal disease, Coronary atherosclerotic heart disease, Percutaneous coronary intervention

## Abstract

**Purpose:**

Dual antiplatelet therapy (DAPT) is the cornerstone for patients with coronary atherosclerotic heart disease (CHD) undergoing percutaneous coronary intervention (PCI) while increasing the risk of bleeding, particularly when combined with gastrointestinal disease (GID). Rivaroxaban 10 mg once daily is widely used in Asia. This study compared the effects of low-dose rivaroxaban (10 mg daily) plus clopidogrel vs. DAPT in CHD patients with GID undergoing PCI.

**Methods:**

In this prospective, single-center, randomized controlled trial, eligible CHD patients with GID undergoing PCI were randomized (1:1) to either the dual pathway inhibition (DPI) group (rivaroxaban 10 mg plus clopidogrel 75 mg daily) or the DAPT group (aspirin 100 mg plus clopidogrel 75 mg daily). The primary outcome was Bleeding Academic Research Consortium (BARC) type 2–5 bleeding. The secondary outcome was major adverse cardiovascular or cerebrovascular events (MACCE), which included cardiac death, nonfatal myocardial infarction, ischemia-driven target vessel revascularization, all-cause death, stent thrombosis, and stroke during the 6-month follow-up.

**Results:**

A total of 1042 patients were enrolled and analyzed (DPI, 522; DAPT, 520). Low-dose rivaroxaban (10 mg daily) plus clopidogrel was non-inferior to DAPT in BARC type 2–5 bleeding [8 (1.5%) vs. 6 (1.2%), absolute risk difference 0.38%, 95% confidence interval (CI) (− 1.02–1.78), *p* < 0.0001 for non-inferiority]. Abdominal pain was significantly lower in the DPI group (*p* = 0.009). Other abdominal discomforts, gastrointestinal bleeding, or MACCE were similar.

**Conclusions:**

In CHD patients with GID undergoing PCI, low-dose rivaroxaban (10 mg daily) plus clopidogrel was non-inferior to DAPT.

**Trial registration:**

Chinese Clinical Trial Registry ChiCTR2100044319. Registered on March 16, 2021.

**Supplementary Information:**

The online version contains supplementary material available at 10.1007/s10557-025-07682-5.

## Introduction

Dual antiplatelet therapy (DAPT) is essential after percutaneous coronary intervention (PCI) but increases bleeding risks [1–3]. More than 10% of patients in China experience aspirin intolerance, including gastrointestinal (GI) discomfort, bleeding, and allergy [4, 5]. Individuals with a history of severe gastrointestinal disease (GID) experience a twofold increase in the annual rates of upper GI complications from chronic low-dose aspirin use compared to the general population, with rates increasing exponentially each decade of age [6, 7]. GI bleeding is the most frequent hemorrhagic complication in patients on DAPT and can lead to DAPT cessation, posing a potentially higher risk of death than myocardial infarction (MI) [8, 9]. It is imperative for coronary atherosclerotic heart disease (CHD) patients with GID undergoing PCI to consider alternative antithrombotic therapies that effectively reduce bleeding and aspirin intolerance while maintaining ischemic benefits.

Dual pathway inhibition (DPI) combines an antiplatelet agent with an anticoagulant, effectively targeting both platelet activity and the coagulation cascade. It has been identified as a potential substitute for long-term DAPT during the stable phase of atherosclerotic cardiovascular disease (ASCVD) in patients at an increased risk of recurrent ischemic events [10]. Rivaroxaban, a widely used direct oral anticoagulant (DOAC), targets activated coagulation factor X. When used with a P2Y_12_ inhibitor, it reduces the risk of major bleeding while maintaining comparable efficacy to DAPT in patients with acute coronary syndrome (ACS) or atrial fibrillation (AF) [11–15]. A standard dose of rivaroxaban 20 mg or 15 mg (in cases of impaired renal function) once daily is commonly used for the prevention and treatment of pulmonary embolism and deep vein thrombosis, as well as for reducing the risk of cardioembolic stroke in patients with AF [16]. Recently, the use of low-dose rivaroxaban (10 mg once daily) has become increasingly common in clinical practice across Asia [17–20]. It has shown comparable risks of thromboembolism and bleeding to standard doses in patients with AF in Asia [21].

This study aimed to evaluate the effects of low-dose rivaroxaban (10 mg once daily) versus aspirin, both with clopidogrel, in CHD patients with GID undergoing PCI, potentially offering an alternative to DAPT for those at high risk of GI bleeding or with aspirin intolerance, thereby improving their prognosis.

## Methods

### Trial Design and Participants

This study was designed as a single-center, open-label, randomized, non-inferiority trial conducted in China (ChiCTR2100044319). The previous publication detailed the rationale and design [22]. Participants were consecutively enrolled from the General Hospital of the Northern Theater Command. Patients included in the study must have met the following eligibility criteria: patients aged between 18 and 75 years, diagnosed with stable CHD, or non-ST-segment elevation acute coronary syndromes (NSTE-ACS) with a Global Registry of Acute Coronary Events (GRACE) score of less than 140 points, in combination with GID. GID was specified as any of the following: acute or chronic gastritis, GI ulcers or bleeding, or gastric mucosal erosion that had healed for a period of 1 to 12 months, any GI dysfunction diagnosed by a specialist, or a GI tumor scheduled for surgery. GI dysfunction referred to functional dyspepsia in our study, defined as symptoms of epigastric pain, postprandial fullness, epigastric burning, and belching caused by disturbances of gastric and duodenal function, excluding any organic disorders that may produce similar symptoms [23]. Patients who took aspirin and experienced adverse effects related to aspirin (e.g., abdominal discomfort, abdominal distension) were also included. ST-segment elevation myocardial infarction (STEMI) or non-ST-segment elevation myocardial infarction (NSTEMI) patients who had a GRACE score greater than 140 points were excluded from the study. Additional detailed inclusion and exclusion criteria are provided in Supplemental file 1.

### Randomization and Treatment

**Fig. 1:**
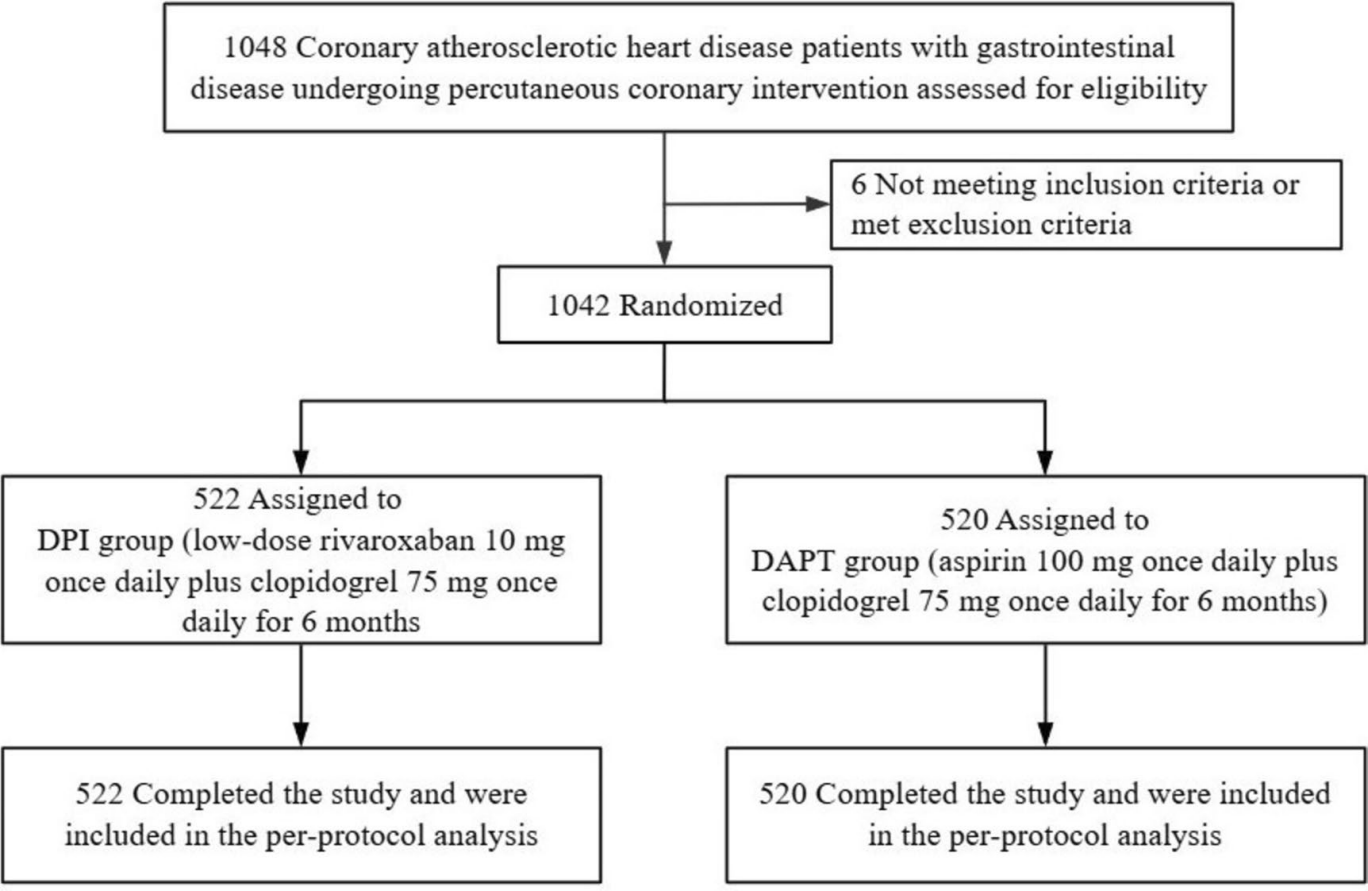
Flow diagram of the study

Written informed consent was obtained from all patients for their participation in the study 1 day before the procedure. A maintenance dose of aspirin (100 mg once daily) plus clopidogrel (75 mg once daily) or ticagrelor (90 mg twice daily) was administered orally at least 12 h before coronary angiography for patients who had routinely taken these medications in the past. For patients who had never taken them or had taken them routinely for less than 5 days before inclusion, a loading dose of aspirin (100–300 mg) plus a loading dose of clopidogrel (300–600 mg) or ticagrelor (180 mg) was administered orally at least 12 h before coronary angiography. After the procedure, all potential participants were assessed for eligibility before randomization. Patients who were unwilling or unable to undergo PCI were not allowed to undergo randomization. Randomization was performed immediately after the completion of PCI. Randomization was performed by using the method of computerized randomization. An independent professional statistician was responsible for generating a random number table using SPSS 25.0 software [24]. Each eligible patient was assigned a random number and was randomly allocated to either the DPI group (rivaroxaban plus clopidogrel) or the DAPT group (aspirin plus clopidogrel) in a 1:1 ratio after the procedure. The study treatment was initiated without delay following randomization. As illustrated in Fig. 1, patients in the DPI group received a daily dose of rivaroxaban at 10 mg, combined with clopidogrel at 75 mg, while patients in the DAPT group received a daily dose of aspirin at 100 mg, combined with clopidogrel at 75 mg. Medications were prescribed continuously for 6 months in both groups. Additional medications were provided at the discretion of the responsible physician, in accordance with standard clinical practice. Patients who were treated with ticagrelor preoperatively discontinued ticagrelor after PCI and received a loading dose of clopidogrel (300 mg) 24 h after the last dose of ticagrelor. A maintenance dose of clopidogrel (75 mg once daily) was administered thereafter.

### Outcomes and Definitions

The primary outcome was Bleeding Academic Research Consortium (BARC) type 2–5 bleeding events. The secondary outcome was major adverse cardiovascular or cerebrovascular events (MACCE), defined as a composite of cardiac death, nonfatal MI, ischemia-driven target vessel revascularization, all-cause death, stent thrombosis, and stroke. Other secondary outcomes included the incidences of each component of MACCE and major bleeding events (BARC type 3–5 bleeding). Detailed outcomes definitions are provided in Supplemental file 2. GI adverse events, including abdominal discomfort (abdominal pain, abdominal distension, sour regurgitation, and ructus) and GI bleeding (haematemesis and melena), were also collected. A standardized scoring table was used to assess GI symptoms. This questionnaire assessed both the severity and frequency of GI symptoms using a five-point scoring system. After randomization, all participants were asked to complete the questionnaire before the start of the trial and again at the 6-month follow-up. The detailed scoring table for GI symptoms is provided in Supplementary file 3. A clinical follow-up was performed at 6 months after randomization. To facilitate the effective follow-up, all patients were thoroughly informed of the significance, duration, and procedures before participating in the study. Additionally, researchers contacted patients by telephone and maintained regular communication with them via instant messaging software, ensuring their continued participation.

All adverse events (AEs) and serious adverse events (SAEs) were systematically collected. An independent Data and Safety Monitoring Board (DSMB) was responsible for evaluating the safety of the study through the regular monitoring of outcome events. All reported events were assessed and classified by members of an independent Clinical Events Committee (CEC). The members of the committee were not provided with any information related to the assignment.

### Sample Size and Statistical Analysis

We estimated that the incidence of BARC type 2–5 bleeding at 6 months was 6% in both the DPI and DAPT groups, based on data obtained from the GEMINI-ACS-1 and OPT-CAD trials [14]. The non-inferiority threshold was set at 4.7% points, representing the minimum absolute difference in the incidence of the primary outcome between the two groups that would be considered non-inferior. On the assumption of a dropout rate of 10%, a sample size of 1020 was calculated to demonstrate non-inferiority (power 85%, α = 2.5%, one-sided). Consequently, there would be 510 patients in each group available for analysis after randomization. The sample size was calculated using the statistical software SAS (Version 9.3).

Continuous variables were presented as either mean ± standard deviation (SD) or median with interquartile range (IQR). Categorical variables were presented as numbers (percentages). All statistical tests were two-tailed, with a *p*-value of less than 0.05 indicating statistical significance, except for the non-inferiority test. All statistical analyses were performed using IBM SPSS Statistics software, version 25.0.

All outcomes and subgroup analyses in the study were performed on a per-protocol set (PPS). The 6-month event analyses of BARC type 2–5 bleeding and MACCE were conducted using time-to-event data. The results were presented in Kaplan-Meier plots and compared using the log-rank test. Subgroup analyses for the primary outcome were performed based on the following factors: sex, age, diabetes status, hypertension, smoking status, history of MI, history of PCI, creatinine clearance (Ccr) level, type of CHD, and the presence of multi-vessel lesions.

## Results

Finally, from April 2021 to May 2023, a total of 1042 patients eligible for randomization were enrolled and assigned to groups: 522 patients were assigned to the DPI group, and 520 patients were assigned to the DAPT group. All 1042 patients completed the follow-up and entered into the final PP analysis (Fig. 1). No patients withdrew from the trial or were lost to follow-up during the study period.

Baseline and procedural characteristics were found to be comparable (Tables 1 and 2). Nearly 39% of the participants were aged 65 years or older. Stent implantation was performed in 91.5% of the patients, while the remaining patients underwent percutaneous transluminal coronary angioplasty (PTCA). Bivalirudin was administered to 666 patients during the operation, including 326 (62.5%) in the DPI group and 340 (65.4%) in the DAPT group. Coronary angiography showed a higher incidence of multi-vessel lesions (59.6%) and left main coronary artery disease (5.6%) in the DAPT group.

**Table 1:** Baseline characteristics of the participants

Characteristics	Total (*N* = 1042)	DPI group (*N* = 522)	DAPT group (*N* = 520)	*p-*value
Demographics				
Age (years)	60.9 ± 8.4	61.4 ± 8.2	60.5 ± 8.5	0.0708
≥ 65 years, no. (%)	406 (39.0%)	214 (41.0%)	192 (36.9%)	0.1776
Men, no. (%)	752 (72.2%)	381 (73.0%)	371 (71.3%)	0.5542
Body mass index, (kg/m^2^)^a^	25.5 ± 3.2	25.4 ± 3.2	25.6 ± 3.3	0.4696
Cardiovascular risk factors, no. (%)				
Hypertension	711 (68.2%)	338 (64.8%)	373 (71.7%)	0.0155
Hyperlipidemia	34 (3.3%)	20 (3.8%)	14 (2.7%)	0.3008
Diabetes mellitus	393 (37.7%)	195 (37.4%)	198 (38.1%)	0.8104
Active smoking	342 (32.8%)	172 (33.0%)	170 (32.7%)	0.9294
Previous medical history, no. (%)				
Previous myocardial infarction	223 (21.4%)	107 (20.5%)	116 (22.3%)	0.4764
Previous percutaneous coronary intervention	380 (36.5%)	185 (35.4%)	195 (37.5%)	0.4899
Previous stroke	192 (18.4%)	93 (17.8%)	99 (19.0%)	0.6108
CHD classification, no. (%)				
Non-ST-segment elevation myocardial infarction	89 (8.5%)	41 (7.9%)	48 (9.2%)	0.4267
Unstable angina	953 (91.5%)	481 (92.1%)	472 (90.8%)	0.4267
Characteristic at admission				
Hemoglobin (g/L)	142.4 ± 13.7	142.4 ± 13.4	142.5 ± 13.8	0.8366
Anemia, no. (%)	22 (2.1%)	10 (1.9%)	12 (2.3%)	0.6564
Platelet count (10^9^/L)	230.2 ± 58.5	233.0 ± 60.7	229.4 ± 57.3	0.3318
Creatinine clearance (ml/min)^b^	102.8 ± 28.4	101.6 ± 27.3	104.1 ± 29.5	0.1509
Left ventricular ejection fraction (%)	60.0 ± 7.0	60.2 ± 6.6	59.8 ± 7.4	0.4101
GRACE score	98.7 ± 21.2	100.2 ± 20.8	97.2 ± 21.5	0.0217
Concomitant medication at discharge, no. (%)				
Statins	1030 (98.8%)	518 (99.2%)	512 (98.5%)	0.2428
ACE inhibitors/ARB	338 (32.4%)	162 (31.0%)	176 (33.8%)	0.3324
Beta-blocker	656 (63.0%)	322 (61.7%)	334 (64.2%)	0.395
Proton pump inhibitor	907 (87.0%)	460(88.1%)	447 (86.0%)	0.299

Data are expressed as mean ± SD, or number (percentage)

*DPI* dual pathway inhibition, *DAPT* dual antiplatelet therapy, *CHD* coronary atherosclerotic heart disease, *GID* gastrointestinal disease, *GRACE* Global Registry of Acute Coronary Events, *ACE* angiotensin-converting enzyme, *ARB* angiotensin receptor blocker

^a^Body mass index was calculated as weight in kilograms divided by height in meters squared

^b^Creatinine clearance was calculated with the use of the Cockcroft–Gault equation

**Table 2:** Procedural characteristics of the participants

Characteristics	Total (*N* = 1042)	DPI (*N* = 522)	DAPT (*N* = 520)	*p*-value
Revascularization strategy				
PTCA, no. (%)	89 (8.5%)	36 (6.9%)	53 (10.2%)	0.057
Stent implantation, no. (%)	953 (91.5%)	486 (93.1%)	467 (89.8%)	0.057
Arterial access site				
Radial, no. (%)	1029 (98.8%)	516 (98.9%)	513 (98.7%)	0.7748
Femoral, no. (%)	11 (1.1%)	5 (1.0%)	6 (1.2%)	0.757
Intraoperative anticoagulant				
Bivalirudin, no. (%)	666 (63.9%)	326 (62.5%)	340 (65.4%)	0.324
Heparin, no. (%)	367 (35.2%)	194 (37.2%)	173 (33.3%)	0.188
Multi-vessel lesions, no. (%)	577 (55.4%)	267 (51.1%)	310 (59.6%)	0.006
Left main coronary artery, no. (%)	45 (4.3%)	16 (3.1%)	29 (5.6%)	0.0461
Number of stents per patient	1.5 ± 0.9	1.5 ± 0.8	1.6 ± 1.0	0.16
Total length of stent (mm)	33.0 (23.0, 51.0)	32.0 (21.25, 47.0)	36.0 (24.0, 54.0)	0.003

Data are expressed as mean ± SD, median and interquartile range (IQR), or number (percentage)

*DPI* dual pathway inhibition, *DAPT* dual antiplatelet therapy, *PTCA* percutaneous transluminal coronary angioplasty

### Primary Outcome

**Fig. 2:**
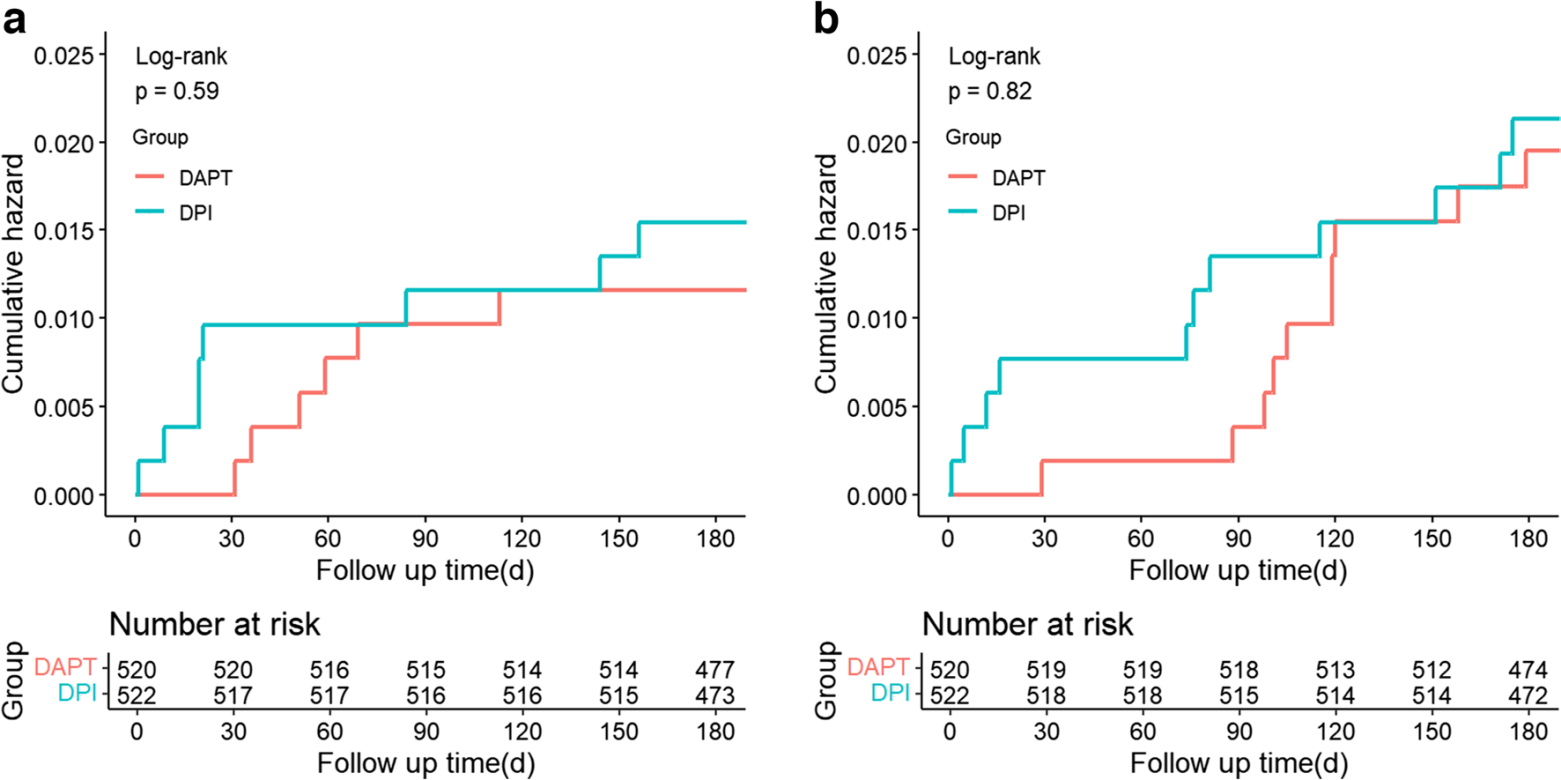
Time-to-event curves for Bleeding Academic Research Consortium (BARC) type 2–5 bleeding through 6-month follow-up. **b** Time-to-event curves for major adverse cardiovascular or cerebrovascular events (MACCE) through 6-month follow-up

There were 8 patients (1.5%) in the DPI group and 6 patients (1.2%) in the DAPT group who experienced BARC type 2–5 bleeding (1.4%). The non-inferiority of low-dose rivaroxaban plus clopidogrel versus DAPT was reached for the primary outcomes [8 (1.5%) vs. 6 (1.2%), absolute risk difference 0.38%, 95% confidence interval (CI) (− 1.02–1.78), *p* < 0.0001 for non-inferiority] (Table 3 and Fig. 2a).

**Table 3:** Results for the primary and secondary outcomes

Characteristics	DPI (*N* = 522)	DAPT (*N* = 520)	HR (95%CI)	*p-*value
Primary outcome				
BARC 2–5* bleeding	8 (1.5%)	6 (1.2%)	1.33 (0.46–3.84)	0.5946
Gastrointestinal	4 (0.8%)	4 (0.8%)	0.59 (0.13–2.77)	0.5049
Gingiva	3 (0.6%)	0 (0.0%)	–	–
Nose	1 (0.2%)	2 (0.4%)	0.62 (0.05–7.00)	0.6975
Secondary outcome				
MACCE	11 (2.1%)	10 (1.9%)	1.10 (0.47–2.60)	0.8238
Other secondary outcomes				
All-cause death	3 (0.6%)	1 (0.2%)	3.00 (0.31–28.81)	0.3418
Cardiac death	0 (0.0%)	1 (0.2%)	–	–
Nonfatal myocardial infarction	1 (0.2%)	0 (0.0%)	–	–
Stroke	3 (0.6%)	1 (0.2%)	2.99 (0.31–28.79)	0.3422
Ischemia-driven target vessel revascularization	3 (0.6%)	9 (1.7%)	0.33 (0.09–1.22)	0.0973
Stent thrombosis	2 (0.4%)	3 (0.6%)	0.67 (0.11–3.99)	0.6576
BARC 3–5 bleeding	1 (0.2%)	4 (0.8%)	0.25 (0.03–2.22)	0.2125

Data are expressed as number (percentage)

*DPI* dual pathway inhibition, *DAPT* dual antiplatelet therapy, *BARC* Bleeding Academic Research Consortium, *MACCE* major adverse cardiac or cerebral events

^*^The absolute rate difference between the two groups was 0.38 percentage point (95%CI, − 1.02–1.78), indicating that rivaroxaban plus clopidogrel was non-inferior to aspirin plus clopidogrel for BARC type 2–5 bleeding (*p-*value for non-inferiority < 0.0001)

### Secondary and Other Outcomes

There were no significant differences in the incidence of MACCE between the two groups [11 (2.1%) vs. 10 (1.9%), hazard ratio (HR) 1.10, 95%CI (0.47–2.60), *p* = 0.8238] (Table 3 and Fig. 2b). The incidence of each component of MACCE, which includes cardiac death, nonfatal MI, ischemia-driven target vessel revascularization, all-cause death, stent thrombosis, and stroke, as well as BARC type 3–5 bleeding, was comparable between the two groups (all *p* > 0.05).

### Analyses for Abdominal Discomfort and Gastrointestinal Bleeding Events

The incidence of abdominal pain was significantly lower in the DPI group compared to the DAPT group (*p* = 0.009). In terms of other types of abdominal discomfort, such as abdominal distension, sour regurgitation, and belching, no significant differences were found between the two groups (all *p* > 0.05). Additionally, with respect to GI bleeding, including haematemesis and melena, no significant differences were found between the two groups (all *p* > 0.05) (Table 4).

**Table 4:** Incidence of abdominal discomfort and gastrointestinal bleeding events

Characteristics	DPI (*N* = 522)	DAPT (*N* = 520)	HR (95%CI)	*p-*value
Abdominal discomfort				
Abdominal pain	25 (4.8%)	50 (9.6%)	0.53 (0.33–0.85)	0.009
Abdominal distension	30 (5.7%)	26 (5.0%)	1.20 (0.71–2.03)	0.492
Sour regurgitation	17 (3.3%)	16 (3.1%)	1.08 (0.55–2.15)	0.816
Ructus	4 (0.8%)	3 (0.6%)	1.40 (0.31–6.27)	0.658
Gastrointestinal bleeding				
Haematemesis	1 (0.2%)	1 (0.2%)	0.99 (0.06–15.85)	0.995
Melena	5 (1.0%)	6 (1.2%)	0.83 (0.23, 2.72)	0.759

Data are expressed as number (percentage)

*DPI* dual pathway inhibition, *DAPT* dual antiplatelet therapy, *BARC* Bleeding Academic Research Consortium

### Subgroup Analyses for the Primary Outcome

**Fig. 3:**
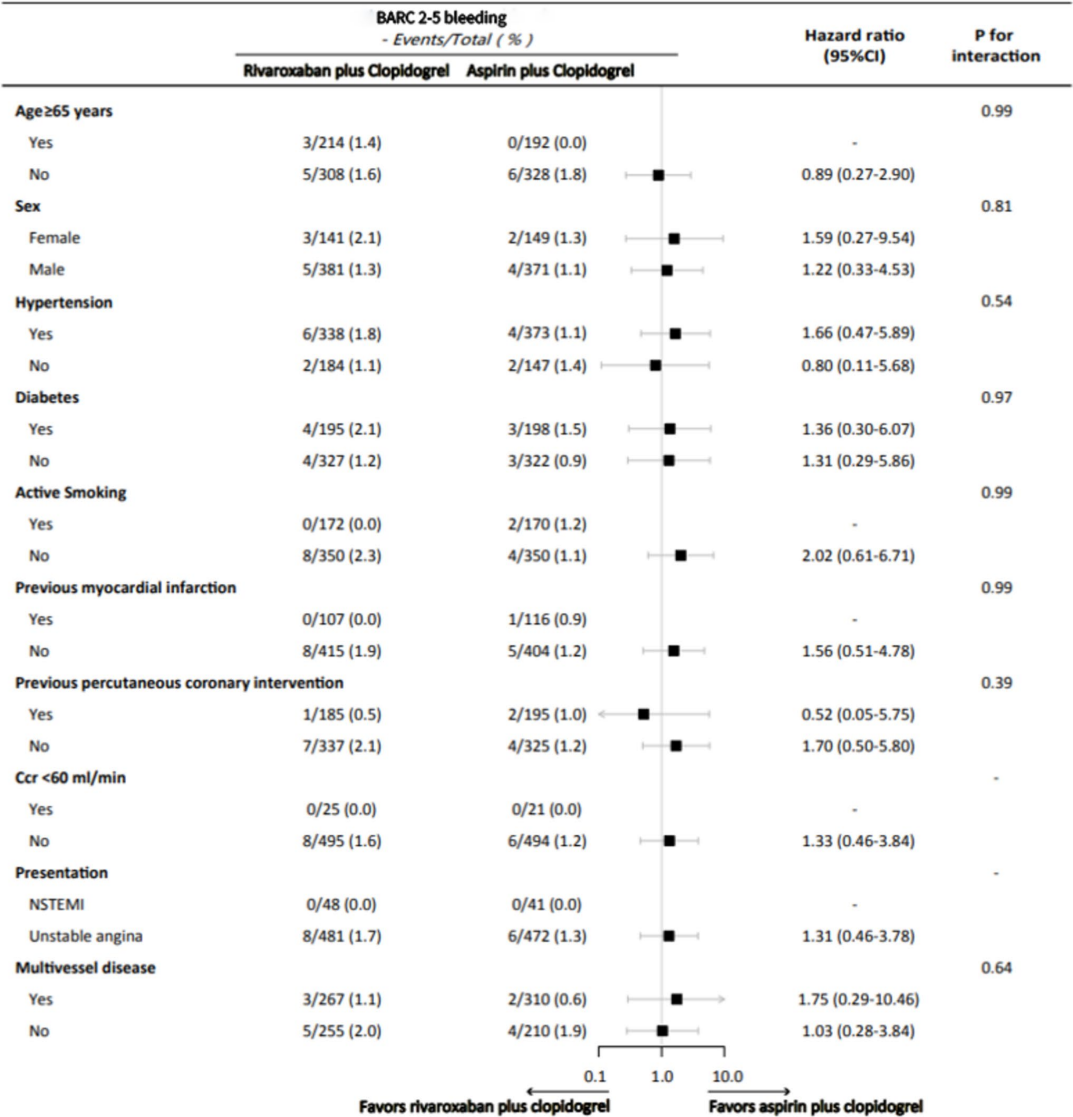
Subgroup analyses for the incidences of Bleeding Academic Research Consortium (BARC) type 2–5 bleeding at 6 months

The treatment effects of low-dose rivaroxaban combined with clopidogrel in the incidence of BARC type 2–5 bleeding were consistent across multiple subgroups. No significant interactions were observed regarding age, sex, hypertension, diabetes status, smoking status, history of MI, history of PCI, Ccr level, type of CHD, and multi-vessel lesions (Fig. 3).

## Discussion

Low-dose rivaroxaban plus clopidogrel was found to be non-inferior to DAPT for BARC type 2–5 bleeding events at 6 months in patients with CHD and GID undergoing PCI. This combination could reduce abdominal pain without increasing GI bleeding or other abdominal discomforts, with no differences in MACCE, its individual components, or BARC type 3–5 bleeding.

Although the use of DAPT after an ACS has reached a consensus in guidelines, patients remain at risk for recurrent cardiovascular events [25]. Adding oral anticoagulants like rivaroxaban or apixaban to DAPT reduces ischemic events but increases bleeding risk in patients with ACS [26–29]. The WOEST trial found that the DPI regimen involving clopidogrel and warfarin resulted in a lower incidence of bleeding in patients who required anticoagulation after PCI compared to triple therapy with clopidogrel, aspirin, and warfarin [11]. Similarly, the PIONEER AF-PCI trial found that a DPI regimen involving clopidogrel and rivaroxaban resulted in a lower risk of clinically significant bleeding compared to triple therapy (clopidogrel, aspirin, and warfarin) in patients with AF [13]. These findings indicate that a threshold has been reached regarding the use of additional antithrombotic therapies in patients with post-ACS or AF, and the risk of bleeding appears to be significantly influenced by the use of aspirin.

The DPI regimen has emerged as a substitute for long-term DAPT for stable ASCVD patients who are at an increased risk of recurrent ischemic events [10]. In the GEMINI-ACS-1 and PIONEER AF-PCI trials, rivaroxaban 2.5 mg twice daily or 15 mg once daily plus a P2Y12 inhibitor was shown to be associated with a comparable or reduced risk of bleeding in patients with ACS or AF [12–14]. Our study differs from these trials by focusing on patients with CHD and GID undergoing PCI, who are at a higher risk for GI bleeding and aspirin intolerance.

This study also provides the first clinical experience with low-dose rivaroxaban in patients with CHD and GID undergoing PCI. Rivaroxaban at a dosage of 20 mg or 15 mg once daily is now a standard practice for the prevention and treatment of pulmonary embolism and deep vein thrombosis, as well as for reducing the risk of cardioembolic stroke in patients with AF [16]. Low-dose rivaroxaban (10 mg) was chosen for several reasons. First, low-dose rivaroxaban (10 mg) is widely used in real-world clinical practice in Asian patients with AF [17–20, 30, 31]. Low-dose rivaroxaban (10 mg daily) was associated with similar risks of thromboembolism and bleeding compared to the standard dose in Asian patients with AF [21]. Second, the dosage of rivaroxaban currently used worldwide is not entirely consistent. The ATLAS ACS-TIMI-51 trial indicated that rivaroxaban at a very low dosage of 2.5 mg may be more beneficial than 5 mg for patients with recent ACS [27]. However, the very low-dose rivaroxaban is not recommended or widely used in Chinese guidelines or real clinical practice. There is controversy about its efficacy, as a previous study suggested that rivaroxaban at a dosage of 2.5 or 5 mg twice daily may not provide the desired therapeutic benefits, indicating that higher doses should be explored in future trials [32]. Third, optimizing the dosage of rivaroxaban should consider factors like age, renal impairment, ethnicity, body mass index, and sex [33]. Rivaroxaban achieves its antithrombotic effect in a concentration-dependent manner. Previous studies involved a higher proportion of Caucasians than Asians. Research indicated that East Asian populations face an increased risk of major bleeding events when undergoing antithrombotic therapies compared to Caucasians [34]. Asians tend to be smaller in stature, and the pharmacokinetic and pharmacodynamic characteristics of most antithrombotic drugs are improved in this population [34].

The incidence of bleeding observed in our study may appear lower than that reported in the PIONEER AF-PCI trial, but it is slightly higher than in the GEMINI-ACS-1 trial, which demonstrated a bleeding risk comparable to that of DAPT in patients with ACS undergoing revascularization. The bleeding incidence was reported at 1% for both groups in the GEMINI-ACS-1 trial, whereas our findings reported incidences of 1.5% and 1.2%. This may be attributed to the following reasons: First, and most obviously, the dose of rivaroxaban used in this study was higher compared to that in the GEMINI-ACS-1 trial (10 mg once daily vs. 2.5 mg twice daily). Second, our study designated BARC type 2–5 bleeding as the primary outcome, whereas the GEMINI-ACS-1 trial focused on BARC type 3a and higher bleeding. In our study, BARC type 2 bleeding accounted for nearly 64% of all BARC type 2–5 bleeding. Third, it may be attributed to the participant characteristics in our study: Chinese CHD patients with GID, along with a larger proportion of females (27.8%), which are high-risk factors for bleeding [34, 35]. It is important to note that while the incidence of BARC type 2–5 bleeding within the DPI group achieved non-inferiority, it was slightly higher than that in the DAPT group. A lower percentage of patients receiving intraoperative bivalirudin in the DPI group (62.5%) compared to the DAPT group (65.4%) might explain this discrepancy. Intraoperative bivalirudin has been shown to reduce bleeding risk in CHD patients compared to heparin, with or without glycoprotein IIb/IIIa inhibitors (GPIs) [36].

To reduce GI intolerance, most guidelines recommend the use of proton pump inhibitors (PPIs) for patients with CHD [1, 37, 38]. However, recent trials have shown limited effectiveness, along with an increased risk of cardiovascular events and GI injuries [39, 40]. Low-dose rivaroxaban plus clopidogrel appeared to have an advantage over DAPT in reducing the incidence of abdominal pain in this study. In terms of the mechanism of action, the adverse effects of aspirin arise from cyclooxygenase (COX) inhibition, which impacts pathways beyond platelets and reduces prostaglandin I_2_ (PGI_2_) generation [41]. In contrast, rivaroxaban, classified as a DOAC, indirectly influences platelet activation but does not affect PG production [42]. It exerts antithrombotic activity by inhibiting the common coagulation pathway and thrombin generation, as well as directly inhibiting activated factor X, which acts as a potent platelet agonist independent of thrombin, triggering platelet activation via PAR-1 [43]. In terms of pharmacokinetics, unlike aspirin, which irreversibly inhibits COX, the antithrombotic effect of rivaroxaban can be quickly recovered due to its short half-life (5–9 h in adults and 11–13 h in elderly patients), potentially reducing the bleeding risk [41]. However, these are positive inferences. The abdominal discomfort symptom was recorded subjectively and was far less convincing than the primary endpoint of BARC type 2–5 bleeding. It remains challenging to determine the true effects of rivaroxaban plus clopidogrel on GI bleeding due to the few positive events.

The results regarding MACCE were consistent with those of previous RCTs evaluating the DPI regimen, including the PIONEER AF-PCI, WOEST, and GEMINI-ACS-1 trials. Although the results showed that rivaroxaban plus clopidogrel was similar to DAPT in reducing MACCE, it was accompanied by lower statistical power due to the few positive events.

## Limitations

There are several limitations to this study. First, although the regimen of low-dose rivaroxaban (10 mg daily) examined in this study is widely used in Asia for patients with AF, it is not currently approved in the guidelines for the treatment of ACS or AF. The decision to adopt a low dose of 10 mg daily in our study was based on several key reasons, as clarified in the discussion. On the one hand, previous studies primarily focused on Caucasian populations. Due to ethnic and regional differences, clinical evidence supporting the use of rivaroxaban at standard doses in Asian patients remains insufficient. On the other hand, a daily dose of 10 mg of rivaroxaban has been widely used in clinical practice in Asia and has shown comparable efficacy to the standard doses of rivaroxaban, which are 15 or 20 mg daily. It is worth noting that although the advantages of very low-dose rivaroxaban in patients with ACS have been shown, the dosage of 2.5 or 5 mg twice daily has not been recommended or widely used in Chinese guidelines or real clinical practice. The safety and efficacy of other different doses of rivaroxaban are expected to be further explored in the future. Second, whereas our results may provide a reference for optimizing antithrombotic therapy in patients with CHD and GID after PCI, the study population was relatively homogeneous, consisting entirely of Chinese patients aged between 18 and 75 years. It may limit the generalizability of the findings to individuals aged over 75 years and of different nationalities and ethnicities, which should be considered when interpreting the results. Third, we chose a non-inferiority margin of 4.7%, in the setting of the event incidence of 6% in the two groups, which may be considered slightly large. Although the primary outcome reached non-inferiority, the statistical power may be limited due to the large non-inferiority margin and the small number of events. The rationale for selecting this margin was based on several factors, including the results observed in previous studies and the necessity to balance statistical precision with practical significance. Nevertheless, this relatively wide margin may result in an overestimation of the true treatment effect. In addition, the number of positive events on outcomes in the study is small with the exception of abdominal discomfort, leading to insufficient statistical power. These factors should be considered when interpreting the findings of our study. Larger, more adequately powered trials are necessary for a definitive assessment of the regimen. Fourth, the follow-up duration for the primary outcome events in our study was 6 months, which is relatively short. It remains to be determined whether a longer follow-up period would result in significant differences in outcomes between the two groups. A longer follow-up period of 1 year is planned for future research to address these questions. Fifth, follow-up information was obtained from hospital records or telephone calls, which may have introduced bias related to patient recall. Finally, the open-label design of the study, in which both patients and physicians were not blinded to the assigned treatment, may have introduced some inherent bias. Nevertheless, an independent clinical events committee was established for the adjudication of all clinical events in an effort to minimize any potential bias.

## Conclusion

To our knowledge, this was the first prospective, single-center, randomized, non-inferiority trial evaluating low-dose rivaroxaban vs. aspirin, in addition to clopidogrel in CHD patients with GID undergoing PCI. Low-dose rivaroxaban combined with clopidogrel was found to be non-inferior to DAPT in BARC type 2–5 bleeding at 6 months. No difference was observed regarding the MACCE, its individual components, or BARC type 3–5 bleeding at 6 months. Low-dose rivaroxaban (10 mg daily) plus clopidogrel could reduce the incidence of abdominal pain without increasing GI bleeding and other abdominal discomfort. Larger, more adequately powered trials are necessary for a definitive assessment of this clinical problem.

## Supplementary Information

Below is the link to the electronic supplementary material.

Supplementary file1 (DOCX 17 KB)

Supplementary file2 (DOCX 17 KB)

Supplementary file3 (DOCX 16 KB)

## Data Availability

The datasets relevant to this study are available from the corresponding author with reasonable request
