# Supplementary material for: Low-Dose Rivaroxaban vs. Aspirin in Addition to Clopidogrel After Percutaneous Coronary Intervention in Coronary Atherosclerotic Heart Disease Patients with Gastrointestinal Disease
Source: Cardiovasc Drugs Ther. 2025 Mar 21;40(1):201–11. doi: 10.1007/s10557-025-07682-5 (PMC12872657; doi:10.1007/s10557-025-07682-5)
Supplement: Supplementary file 1 — Supplementary file1 (DOCX 17 KB) [file 10557_2025_7682_MOESM1_ESM.docx]

**Supplemental file 1. Detailed inclusion and exclusion criteria**

| **Inclusion criteria** |
| --- |
| 1. 18 to 75 years of age 2. Stable Coronary artery disease or NSTE-ACS with GRACE score < 140 points 3. Gastrointestinal diseases (GID was defined as acute or chronic gastritis, gastric mucosal erosion, gastrointestinal bleeding, or gastrointestinal ulcers that had healed for 1-12 months, gastrointestinal dysfunction diagnosed by a specialist or a gastrointestinal tumor scheduled for surgery) 4. Taking aspirin with a stomachache, abdominal distension, and other discomforts but can tolerate 5. Informed consent |
| **Exclusion criteria** |
| 1. NSTE-ACS with GRACE score > 140 points or STEMI 2. History of intracranial hemorrhage within one month or vital organ bleeding 3. Platelet count < 100 × 10^9^/L 4. Hemoglobin < 100 g/L 5. Active hepatitis or ALT, AST values > 3 × the upper limit of normal 6. Severe renal insufficiency (CrCl < 30 mL/min) 7. Body weight < 45 kg 8. History of aspirin or rivaroxaban allergy 9. Not willing to undergo PCI 10. Severe progressive disease (e.g., malignant tumor) 11. Anticipated life expectancy < 6 months 12. Pregnancy, breastfeeding, and childbearing plans 13. Long-term use of (N)OAC 14. Long-term use of strong CYP3A4 inhibitors or P-glycoprotein inhibitors 15. Long-term use of moderately potent CYP2C19 inhibitors or CYP2C19 inducers 16. Expected to be unable to tolerate medication for 6 months 17. Unable to obtain an informed consent or the researchers think it is not suitable to participate in this trial in any case 18. Participating in other ongoing clinical studiesOral anticoagulants (rivaroxaban, warfarin, etc.) used within 2 weeks |

GRACE Global Registry of Acute Coronary Events, NSTE-ACS non-ST-segment elevation acute coronary syndromes, STEMI ST-segment elevation acute myocardial infarction, ALT alanine aminotransferase, AST aspartate aminotransferase, CrCl creatinine clearance (CrCl = [(140-age) × weight (kg) × (0.85 female)]/[0.818 × Scr (mol/L)], PCI percutaneous coronary intervention, NOAC new oral anticoagulant
