# Supplementary material for: Low-Dose Rivaroxaban vs. Aspirin in Addition to Clopidogrel After Percutaneous Coronary Intervention in Coronary Atherosclerotic Heart Disease Patients with Gastrointestinal Disease
Source: Cardiovasc Drugs Ther. 2025 Mar 21;40(1):201–11. doi: 10.1007/s10557-025-07682-5 (PMC12872657; doi:10.1007/s10557-025-07682-5)
Supplement: Supplementary file 3 — Supplementary file3 (DOCX 16 KB) [file 10557_2025_7682_MOESM3_ESM.docx]

**Supplemental file 3. The scoring table for gastrointestinal symptoms**

There are four categories of symptoms: abdominal pain, abdominal distension, sour regurgitation, ructus. A five-point scoring system was used for each symptom (degree and frequency were scored respectively; Maximum score =4+4=8).

| **Score (s)** | **Symptom** (Maximum score =4) | **Frequency** (Maximum score =4) |
| --- | --- | --- |
| 0 | No symptoms | No seizure |
| 1 | Mild, symptoms are mild, It takes attention to feel it | The episodes occurred 1 day per week |
| 2 | Moderate, subjective symptoms are obvious, but do not affect work and life | The episodes occurred 2-3 days per week |
| 3 | Severe, subjective symptoms are obvious, affecting work and life | The episodes occurred 4-5 days per week |
| 4 | Extremely severe, subjective symptoms are obvious, seriously affecting work and life | It occurs almost daily or continuously |
